# Identification of rare maternal copy number variants by genome-wide analysis of noninvasive prenatal screening data in 113,017 pregnant women

**DOI:** 10.1080/07853890.2026.2721711

**Published:** 2026-09-09

**Authors:** Liang Hu, Xianzhen Cao, Shuxian Zeng, Lijuan Wen, Jiatong Zhong, Yanan Liu, Weiqiang Liu, Fengxiang Wei

**Affiliations:** a Central Laboratory, Maternity & Child Healthcare Hospital of Longgang District, Shenzhen City (Longgang Maternity and Child Institute of Shantou University Medical College), Shenzhen, China; b Longgang District Key Laboratory for Birth Defects Prevention, Shenzhen, China; c Guangdong Engineering Technology Research Center of Genomics and Precision Imaging, Shenzhen, China

**Keywords:** Non-invasive prenatal screening, non-invasive prenatal testing, maternal copy number variant, carrier screening

## Abstract

**Objectives:**

Knowledge of copy number variants (CNVs) is relevant to maternal and fetal health and can be obtained from noninvasive prenatal screening (NIPS) of pregnancy. However, genome-wide analysis of maternal CNVs using NIPS data has not been conducted in large populations.

**Methods:**

For CNV analysis, the human genome was segmented into 10 kilobase pairs (Kb) bins, and the relative sequencing depth of each bin was calculated. The circular binary segmentation algorithm was used to estimate CNVs. Detected CNVs from two pregnancies of the same participant were compared to validate the reproducibility. All CNVs were merged into CNV regions (CNVRs) to evaluate their frequency, distributions, and relationship with disease-related genes and regions.

**Results:**

In this study, 113,017 pregnant women were recruited. A total of 363,886 CNVs larger than 50 Kb were detected in 101,779 individuals and merged into 43,005 CNVRs. For evaluating the reproducibility of CNVs, 90.18% of deletions and 88.07% of duplications were consistent. In general, 78.13% of individuals carried CNVRs that overlapped protein-coding genes, while 14.76% overlapped OMIM genes. We detected 246 novel CNVRs, 134 (54.47%) involving protein-coding genes. For the perspective of maternal-fetal health, we identified 4,984 (4.41%) individuals as carriers of 5,243 CNVs containing known pathogenic or likely pathogenic regions, including 22q11.2 region and DMD gene..

**Conclusions:**

NIPS sequencing data is a reliable source for maternal CNV detection. These CNVs constitute an integrate component in maternal-fetal health management.

## Introduction

Noninvasive prenatal screening (NIPS) performs genome-wide massively parallel sequencing of cell-free DNA (cfDNA) from maternal plasma at low coverage, and has been validated as a reliable method for screening for fetal trisomy 21, 18, and 13 [1]. In addition, NIPS provides information relevant to fetal or maternal chromosomal and subchromosomal variants that have clinical values in diagnosing fetal or maternal chromosomal aneuploidies and significant imbalance variants [2]. This knowledge has broader and essential health implications, including those related to fetal developmental abnormalities and intellectual disability [3]. Several studies have attempted to investigate these genomic abnormalities using the NIPS genome-wide sequencing data [4,5]. However, most of these studies have focused on fetal variants using small cohorts and have not investigated maternal copy number variants (CNVs) [6–9].

While most maternal CNVs derived from NIPS are benign or have unknown clinical significance, some of them may have health implications [10,11]. For example, low penetrance CNVs inherited from unaffected mothers may have a potential clinical impact on the fetus. Although prenatal CNVs and other chromosomal abnormalities can be obtained from cell-free DNA sequenced by NIPS, the maternal cfDNA comprises the majority of cfDNA [12], supporting the feasibility of identifying maternal genomic variants during NIPS. The simultaneous derivation of fetal and maternal CNVs has recently been confirmed by calculating the sequencing depth of segmented chromosomes [9,13]. Several studies have shown that most maternal CNVs detected by NIPS are non-pathogenic or of low penetrance [13,14]. However, this is not surprising considering the low sequencing coverage (0.05–0.2×) in NIPS and the limited sample size (less than 10,000 individuals) of the cohorts used in these studies [13,14]. As expected, only a few maternal CNVs (0.04–0.15 CNVs per individual) were detected in these studies, and most of them were considered background noise of NIPS. Thus, it’s evident that large cohorts are needed to overcome the low coverage sequencing limitation to obtain in-depth knowledge of maternal genomic variants. This knowledge will improve pregnancy management concerning both fetal and maternal health. In addition, this genetic information will expand the existing data sources of human genetics [15].

In this study, we report a thorough analysis using a large cohort of NIPS data covering 113,017 pregnant individuals. Methods were developed to detect rare maternal CNVs using NIPS data. We created a CNV region (CNVR) map for pregnant women and investigated the distribution of CNVRs in the maternal genome and their association with maternal and fetal health.

## Methods

### Participants selection

In this retrospective study, pregnant women who underwent NIPS in Maternity & Child Healthcare Hospital of Longgang District, Shenzhen City from November 2017 to April 2022 were recruited. The raw sequencing data in fastq format and electric medical record was collected for CNV analysis. Benefited from a local public perinatal healthcare fund, every family is available to choose NIPS as the first-tier method for prenatal aneuploidy screening after 12 weeks of gestation. NIPS was implemented in the general population of pregnant women regardless of maternal age, risk of first trimester serum screening and fetal ultrasound findings. Over 65% of local pregnant women were covered under this screening strategy annually. Participants were excluded if: (1) one of the couples carried a confirmed chromosomal aberration, Mendelian inheritance disease, or fetal structural abnormality requiring prenatal diagnosis; (2) triplet or multiple fetus pregnancy. (3) received allogeneic blood transfusion within one year or immunotherapy with exogenous DNA within four weeks or received transplantation or stem cell therapy; (4) with maternal malignant neoplasm.

We removed samples with low sequencing quality, defined as reads that amounted to less than 3.5 million or G and C base content greater than 42%. Therefore, a total of 113,017 Chinese pregnant women who received NIPS in our hospital between November 2017 and October 2022 were included in this study. If a participant had more than one NIPS result in different pregnancies in the past five years, the data of each NIPS were compared, but only the most recent one was recorded. Information on the participants’ maternal age, gestational age, and place of birth was also collected. Each participant was informed and aware of the potential unanticipated findings in this study. Written informed consent was obtained from all participants. The study was conducted by the Declaration of Helsinki of 2013 and approved by the Research Ethics Committee of Maternity & Child Healthcare Hospital of Longgang District, Shenzhen City (Ethics ID: KYXM-2023-037-01; HGRAC ID: CJ1455).

### NIPS and CNVs analysis

The protocol for NIPS was based on the BGI NIFTY chromosomal abnormality test kit (BGI, China) as described previously [16]. A total of 5 mL peripheral blood was collected from each participant using EDTA anticoagulant tubes. Samples were centrifuged at 3,500 rpm for 10 min at 4 °C, and the upper plasma fraction was collected for library preparation. The cfDNA was extracted from plasma by a magnetic bead-based method using the Nucleic Acid Extraction Kit (BGI, China) on the MGI-960 system (BGI, China). Automated library preparation was performed on the MGI-960 system with the Fetal Chromosome Aneuploidy Detection Kit (BGI, China). No fetal cfDNA enrichment method was used for cfDNA extraction and purification. After library preparation, cfDNA was single-end sequenced on the SEQ500 platform (BGI, China) with a read length of 35 bp and an average of 9.7 million reads per sample. To estimate CNVs, all reads were aligned to the human genome reference GRCh37(hg19) using Subread (v2.0.4), and duplicates were removed using Samtools (v1.17) [17,18]. Bedtools (v2.31.0) was used to partition the approximately 3095.71 million base pair (Mb) human genome into 10 kilobase pair (Kb) windows, corresponding to a total of 309,581 windows genome-wide [19]. Read counts were normalized for each window and recalibrated using a locally weighted scatterplot smoothing (LOESS) algorithm to reduce GC content bias. CNVs were then predicted using the circular binary segmentation (CBS) algorithm [20]. To avoid false positives, the resolution of CNVs was limited to 50 Kb due to window size constraints. The breakpoints of the CNVs were also approximated to be 10 Kb steps. Calling of CNVs was avoided in centromere regions of all chromosomes, satellite regions of D and G group chromosomes, and heterochromatin regions of chromosomes 1, 9, and 16, as well as in the human leukocyte antigen (HLA) region at 6p21.3 due to significant polymorphism. The fetal fraction for each NIPS sample was calculated using HALOS software (BGI, Shenzhen, China) [21].

Given the challenges in determining the fetal or maternal origin of cfDNA, we excluded individuals with a fetal fraction greater than 50% to obtain samples predominantly composed of maternal DNA.

### Validation of CNVs

CytoScan 750K Single nucleotide polymorphism (SNP) array (Affymetrix, USA) was used for validation of maternal CNVs in randomly selected individuals. Furthermore, to validate the CNVs for more participants, we compared the CNVs calculated from two independent pregnancy datasets of the same woman in this five-year study period. This validation method assumes that a pregnant woman’s genome remains stable and that no somatic variants induced during two pregnancies. The influence of fetal cfDNA was also excluded from this validation method.

### Annotation of CNVRs

Individual CNV calls were combined into CNVRs by merging fragments with more than 50% overlapped [22]. A unique CNV was directly identified as a CNVR. The size, location, and frequency distribution of CNVRs were analyzed to create a CNVR map. This map was then compared with existing datasets in the dbVar (https://www.ncbi.nlm.nih.gov/dbvar), Decipher, and ClinVar databases (study IDs: nstd174, nstd183, and nstd102, respectively [Supplementary methods]) [23–25]. Novel CNVRs were defined as no more than 50% overlap with any datasets and with a frequency >0.01%. The affected genes of each CNVR were listed according to the gene sets of RefSeq (https://www.ncbi.nlm.nih.gov/refseq/) and Online Mendelian Inheritance in Man (OMIM) databases (https://www.omim.org/). The CNVRs involved dosage-sensitive genes with a score of 3 was interpreted as dosage-sensitive CNVRs [26]. The clinical significance of CNVRs was analyzed using the gene set consisting of 113 genes indicated in the American College of Medical Genetics and Genomics (ACMG) guideline for carrier screening during pregnancy [27]. We selected deletions overlapping genes recommended for screening by the ACMG carrier screening guidelines and duplications that spanned triplosensitive genes as candidate CNVRs with potential clinical significance. Furthermore, the *DMD* gene and 22q11.2 region were selected for detailed analysis of distribution, frequencies and pathogenicity of maternal CNVs.

### Statistical analysis

R v4.3.0 (https://www.r-project.org/) was used for data analysis and plotting. Categorical variables were summarized as frequencies and percentages. For continuous variables, skewness and kurtosis were calculated to evaluate approximate normal distribution, and corresponding statistical description was adopted according to the normality assessment results [28]. Approximately normal distributed variables were expressed as mean ± SD, while non-normal distributed variables were presented as median, minimum and maximum. Independent sample *t*-tests and Wilcoxon tests were performed to compare differences between Gaussian-distributed data and non-Gaussian-distributed data, respectively. The frequencies of duplications and deletions in each 10 Kb bin of chromosomes were visualized using the R package circlize [29]. The CBS algorithm was implemented using the R package DNAcopy (v1.70.0) [30]. In this study, scatter plots were adopted to visualize the raw signal distribution of CNVs as well as deletion and duplication information within each 10-kilobase (10k) bin. Histograms were used to display CNV size distribution, frequency distribution, and the comparison results between dual gestational samples. Bar plots were applied to exhibit the frequencies of high-frequency CNVRs. CNVR distribution maps were utilized to characterize the chromosomal locations of CNVRs, with the code available on Github (https://github.com/miyahappy/nipt_call_cnv).

## Results

### Profiling of maternal CNVs

The mean maternal and gestational ages were 29.5 ± 4.6 years and 117.8 [72,279] days, respectively. The distribution of maternal age, gestation age and fetal fraction was shown in Supplementary Figure S1(a–c). Among all participants, 84.18% (95,138/113,017) were from South China, and 34.34% (38,810/113,017) were from East Guangdong Province, mainly inhabited by Hakka and Chaoshan people (Supplementary Figure S1(d)). The average sequencing depth of the samples was at 0.15× coverage approximately, with an average fetal fraction in cfDNA of 9.46 ± 3.88%.

**Figure 1.**
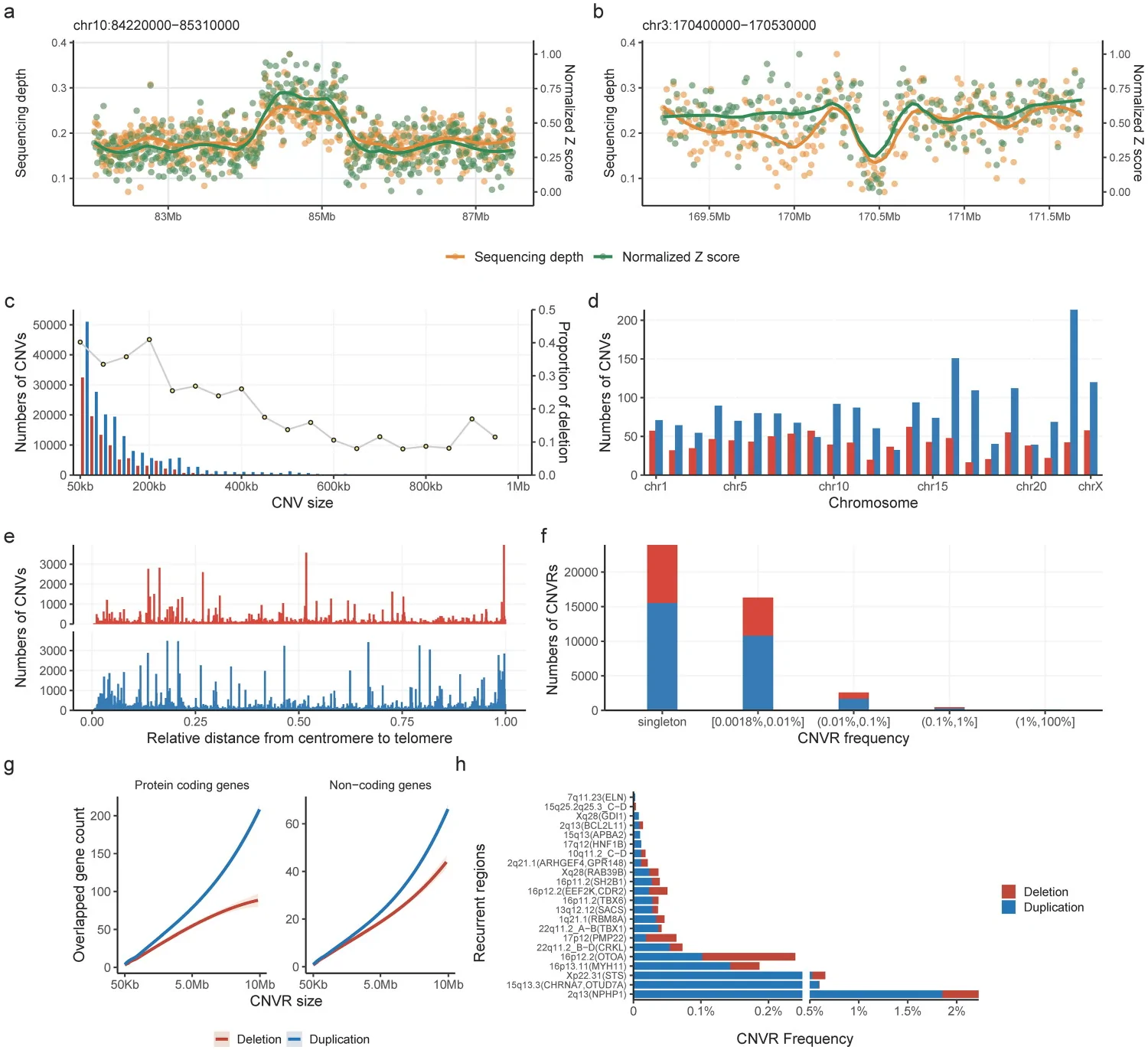
Profiles of maternal copy number variants (CNVs) and CNV regions (CNVRs) detected using prenatal cell-free DNA sequencing data. (a,b) Sequencing depth and normalized *Z*-scores of segmented bins in two examples. Each point presents a 10 kilobase pair (Kb) bin. The smoothed lines are fitted using locally estimated scatterplot smoothing (LOESS) algorithm. (c) Distribution of CNV sizes. (d) CNVs per million base (Mb) pair in each chromosome. (e) CNVs distribution from the centromere to the telomere, which is scaled from zero (centromere) to one (telomere). (f) Frequencies of duplication and deletions. (g) Protein-coding and non-coding genes overlapped by 50 Kb to 10 Mb CNVRs. (h) Duplications and deletions in the top 20 frequent recurrent regions.

After removing low-quality data, CNVs were called after normalizing the sequencing depth by z-scores. The relationship between sequencing depth and its normalization to CNV calling is illustrated in Figure 1(a,b). Normalization improved the specificity of CNV calling. As expected, CNV frequency was negatively correlated with CNV length for duplications and deletions (Figure 1(c)). The chromosomal distribution of the CNVs indicated that duplications located in chromosome 22 most frequently (Figure 1(d)). The chromosomal distribution of CNVs is shown in Figure 1(e). Interestingly, we found that a higher duplication density occurred near the telomere and centromere within individual chromosomes.

**Figure 2.**
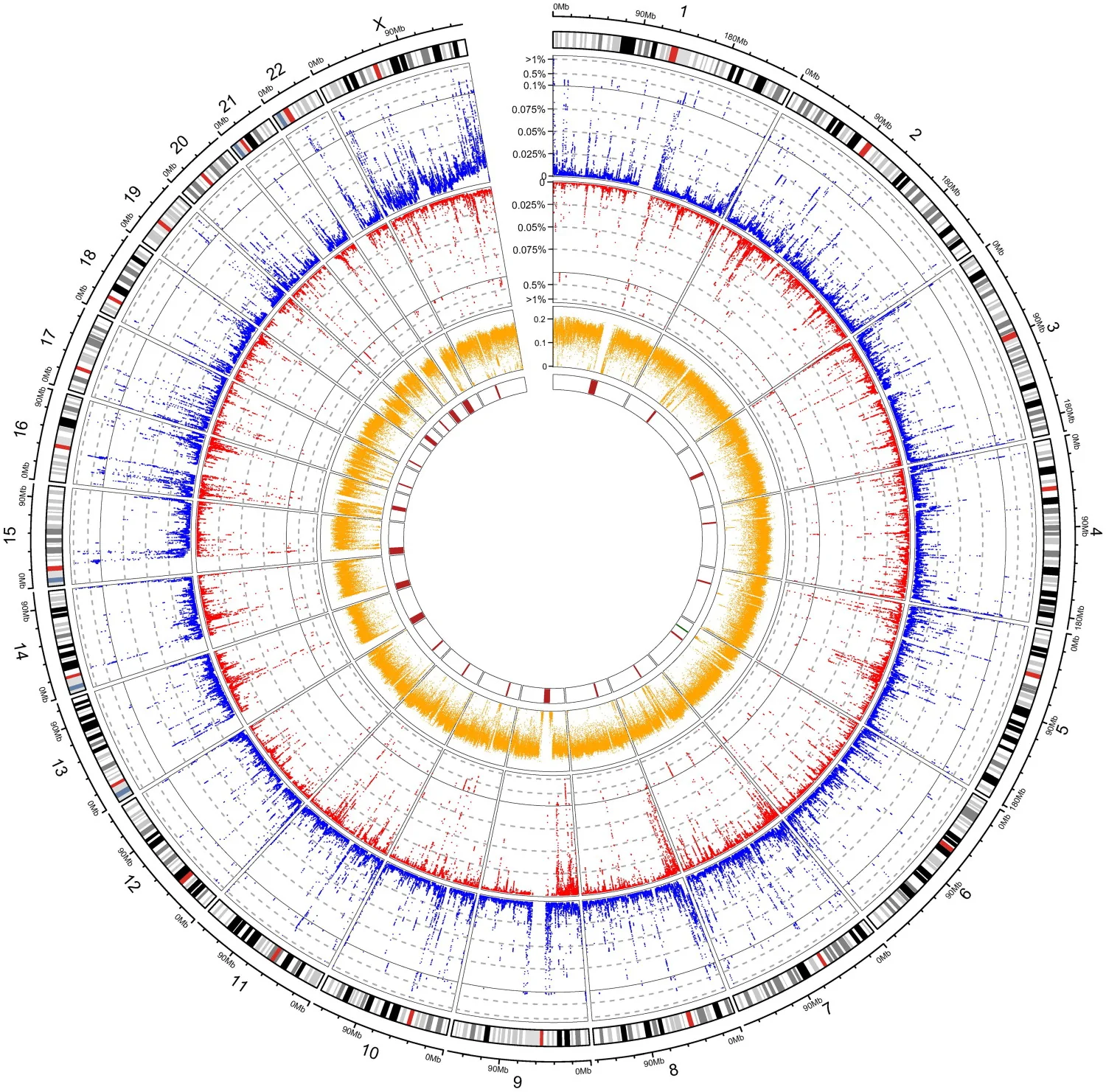
Circle plot of frequencies and sequence depth of each 10 kilobase pair (Kb) bin in the genome. The tracks from the outside to the inside are (1) The ideogram of chromosome 1–22 and chromosome X. (2) Duplication frequencies of each 10 Kb bin. (3) Deletion frequencies of each 10 Kb bin. (4) Sequence depth of each 10 Kb bin. (5) Masked regions without CNV analysis.

A total of 363,886 CNVs larger than 50 Kb were detected in 101,779 individuals, resulting in a positive detection rate of 90.06% (101,779/113,017) and an overall average of 3.22 CNVs per individual. Among all CNVs, 128,296 deletions (1.14 per individual) and 235,590 duplications (2.08 per individual) were detected, ranging in size from 50 Kb to 19.96 Mb, with a median size of 100 Kb. CNVs covered 87.10% (2.7/3.1 Gb) of the genome, with deletion coverage of 51.61% (1.6/3.1 Gb) and duplication coverage of 83.87% (2.6/3.1 Gb). Due to the limitations of the overall sequencing depth and the influence of fetal DNA, the exact copy number or gene coordinates of each CNV was difficult to be determined. The frequency and sequencing depth of CNVs per 10 Kb across the genome and the regions masked from CNV detection are shown in Figure 2.

### Reproducibility of CNVs

A total of five individuals were validated using CytoScan 750K SNP array. The maternal CNVs detected by NIPS and SNP array were compared in Table 1. In 11 CNVs from NIPS data, eight were confirmed by SNP array, with a true positive rate of 72.73% (8/11). Three small duplications range from 50 Kb to 60 Kb were not reproducible using SNP array, indicating the increased possibility of false positive calls for small CNVs.

**Table 1.** Validation of CNVs in five individuals using SNP array.

Patient ID	NIPS			SNP array	
	CNV location	Size (Kb)	CNV type	CNV location	Size (Kb)
1	Chr13:102470000_105580000	3120	Duplication	13q33.1q33.2(102461138_105576766) × 3	3115.63
2	Chr7:63760000_63960000	210	Duplication	7q11.21(63767835_63959000) × 3	191.17
2	Chr9:490000_560000	80	Deletion	9p24.3(495125_565762) × 1	70.64
2	Chr10:135240000_135370000	140	Duplication	10q26.3(135252932_135426386) × 3	173.45
2	Chr15:88850000_90280000	1440	Duplication	15q25.3q26.1(88846127_90288164) × 3	1442.04
2	Chr16:35020000_35070000	60	Duplication	16p11.1(35015046_35069774) × 3	54.73
3	Chr8:11970000_12020000	60	Duplication	Not detected	—
4	Chr7:65410000_65450000	50	Duplication	Not detected	—
5	Chr4:62620000_62730000	120	Deletion	4q13.1(62619788_62730657) × 1	110.87
5	Chr13:22630000_24430000	1810	Deletion	13q12.11q12.12(22639070_24439550) × 1	1800.48
5	Chr13:85530000_85580000	60	Duplication	Not detected	—

CNV, copy number variant; SNP, single nucleotide polymorphism; NIPS, noninvasive prenatal screening.

**Figure 3.**
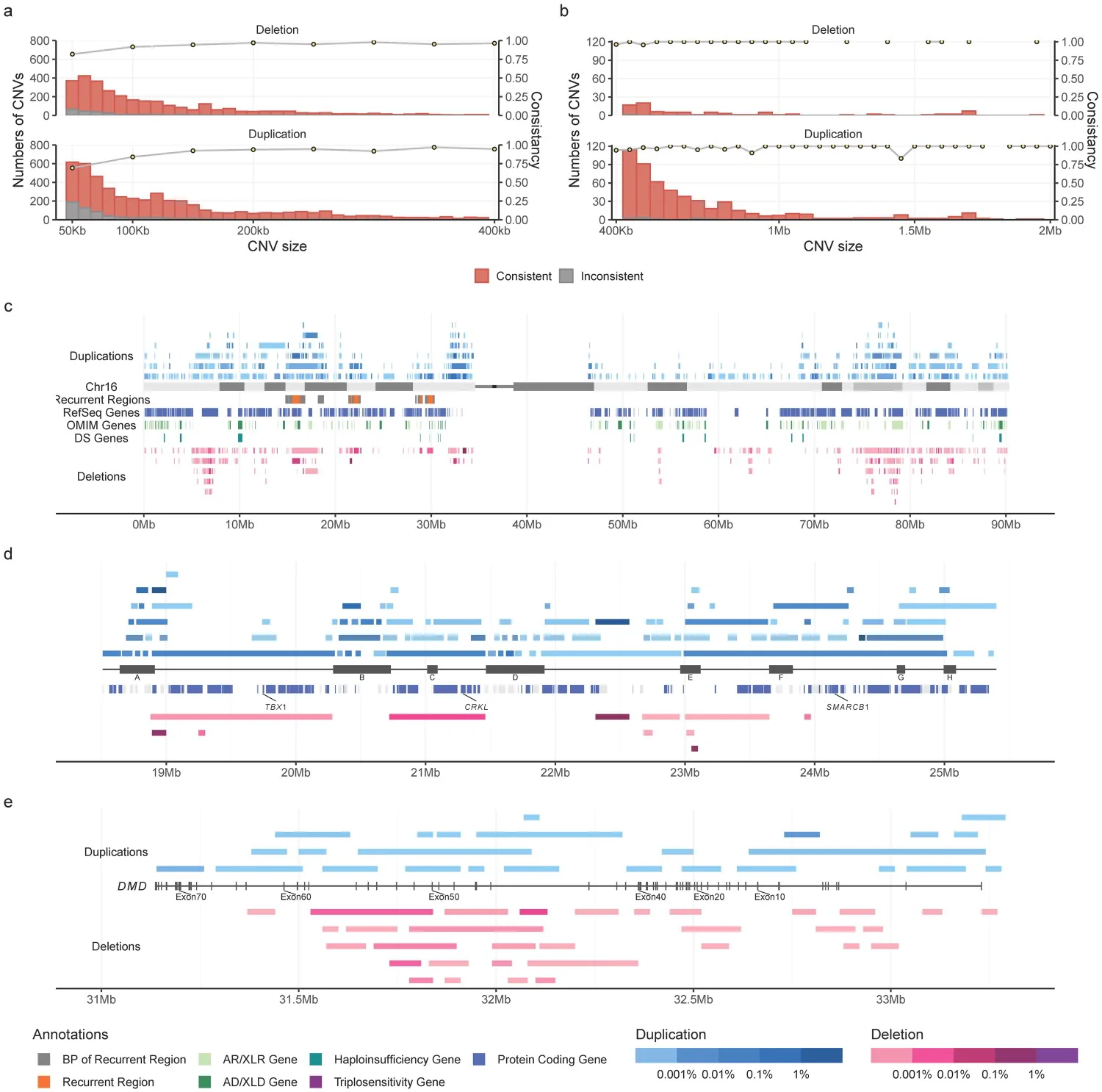
The reproducibility of CNVs and examples of CNV map at the chromosomal, sub-chromosomal and genic levels. (a,b) Consistency of CNVs in two independent pregnancies of same women. The height of bars presents the number of reproducible and unreproducible CNVs in two pregnancies. The lines indicate the consistency rate at different CNV size. (c) CNVR map of chromosome 17. Each CNVR presented as a rectangle, with left and right borders indicating the upstream and downstream breakpoints. (d) CNVRs in 22q11.21 region. (e) CNVRs in the *DMD* gene.

In our cohort, 5,172 women were pregnant twice during the study period. We compared the CNV calculated from the NIPS data in two independent pregnancies. A total of 92.31% (2,915/3,158) of deletions and 87.74% (4,776/5,443) of duplications found in the first pregnancy were repeatedly detected in the second pregnancy, resulting in an overall reproducibility rate of 89.42% (7,691/8,601). A critical factor affecting the reproducibility of CNVs was the size of CNVs. Consistency of CNVs in two independent pregnancies of same women were shown in Figure 3(a,b). For CNVs larger than 100 Kb, 95.88% (1,466/1,529) of deletions and 93.75% (2,985/3,184) of duplications were detected in both pregnancies. However, for deletions and duplications between 50 Kb and 100 Kb, 88.95% (1,449/1,629) and 79.28% (1,791/2,259), were validated in the second pregnancy, respectively. These non-reproducible CNVs were not distributed in any particular hotspot on chromosomes. In addition, some non-reproducible duplications may be false positives caused by lower sequencing depth (Supplementary Figure S1(e)).

### Mapping of CNVs into CNVRs and overlapping regions

All 363,886 CNVs were merged into 43,005 CNVRs, including 35.11% (15,097/43,005) deletions and 64.89% (27,908/43,005) duplications, of which 0.81% (348/43,005) regions were mixed with deletions and duplications. The population frequency distribution of CNVRs was shown in Figure 1(f). Of all CNVRs, 99.90% (42,961/43,005) are rare CNVRs (frequency < 1%). A total of 55.56% (23,895/43,005) CNVRs were detected uniquely in the population, while 7.05% (3,030/43,005) CNVRs had a frequency of greater than 0.01%. CNVRs with frequency over 1% were shown in Supplementary Table S1.

We then mapped CNVRs to individual chromosomes, and illustrated chromosome 16 as an example in Figure 3(c). The complete CNVR distribution maps of all chromosomes were shown in Supplementary Figures S2–S6. CNVRs and their association with pathogenic/likely pathogenic regions, recurrent regions, dosage-sensitive regions, RefSeq genes, OMIM genes, and carrier screen genes in individual chromosomes were also mapped and summarized (Table 2). In general, we identified 4.41% (4,984/113,017) of individuals in our cohort carrying 5,243 known pathogenic or likely pathogenic CNVs in 549 CNVRs (Supplementary Table S2), and 6.56% (36/549) of these CNVRs were located in recurrent regions (Supplementary Table S3). The duplications and deletions in these CNVRs within these recurrent regions will likely affect genes with essential functions and thus may have health consequences.

**Table 2.** Summary of CNVs and CNVRs that overlapped phenotype-associated regions and genes.

Regions and genes overlapped	Individuals		CNV events		CNVRs	
	Count	Percent (%)	Count	Percent (%)	Count	Percent (%)
Known pathogenic/likely pathogenic regions	4,984	4.410	5,243	1.441	549	1.277
Recurrent regions	5,760	5.097	6,144	1.688	36	0.084
Dosage-sensitive regions	589	0.521	589	0.162	50	0.116
RefSeq genes						
Protein coding genes	91,358	80.836	202,886	55.755	25,219	58.642
Non-protein coding genes only	4,805	4.252	49,392	13.573	4,641	10.792
Total	96,163	85.087	252,278	69.329	29,860	69.434
OMIM genes	16,678	14.757	24,803	6.816	3,219	7.485
OMIM XLR genes	1,477	1.307	2,405	0.661	198	0.460
OMIM AD/XLD genes						
Intragenic duplication	2,586	2.288	2,626	0.722	524	1.218
Intragenic deletion	416	0.368	418	0.115	158	0.367
Whole gene loss	438	0.388	603	0.166	165	0.384
Whole gene gain	1,656	1.465	2,754	0.757	416	0.967
Total	5,096	4.509	6,401	1.759	1,263	2.937
Novel CNVs/CNVRs (non-singleton)	6,371	5.637	6,560	1.803	246	0.572
Dosage-sensitive genes						
HI gene	580	0.513	580	0.159	188	0.437
TI gene	48	0.042	48	0.013	16	0.037
Carrier screening genes	1,065	0.942	1,065	0.293	285	0.663

CNV, copy number variant; CNVR, copy number variant region; OMIM, Online Mendelian Inheritance in Man; AR, autosomal recessive inheritance; AD, autosomal dominant inheritance; XLR, X-linked recessive inheritance; XLD, X-linked dominant inheritance; HI, haploinsufficiency; TS, triplosensitivity.

In addition, 80.84% (91,358/113,017) of individuals carried CNVRs that overlapped protein-coding genes, and 14.76% (16,678/113,017) carried CNVRs that overlapped OMIM genes. The Figure 1(g) illustrates the number of genes covered by CNVRs. Deletions affected fewer protein-coding and non-coding genes than duplications. CNVRs in 20 most frequent recurrent regions was shown in Figure 1(h). We found that 4.51% (5,096/113,017) of individuals were carriers of CNVRs involving dominant genes. Considering inheritance and the impact of structural abnormalities on gene function, most CNVRs were benign and did not involve dosage-sensitive genes. *STS* (0.135%, 153/113,017), *DMD* (0.082%, 93/113,017), and *PMP22* (0.045%, 51/113,017) are the most frequently affected dosage-sensitive genes detected in this study (Supplementary Table S4). Furthermore, 0.94% (1,065/113,017) of the individuals in this study were carriers of CNVRs overlapping the genes recommended for carrier screening. Deletions overlapping *DMD*, *HBB*, *PCDH15* and *F8* were most frequent in the pregnant population, which may need further prenatal genetic counselling and laboratory validation (Table 3). In this study, none of the variant carriers were notified of their CNV findings, and no downstream clinical testing was performed for these individuals.

**Table 3.** Top frequent carrier screening genes overlapped by NIPS revealed maternal CNVRs.

Gene (OMIM ID)	Location	Main phenotype	Inheritance	Deletions		Duplications		Total
				Carriers	Frequency	Carriers	Frequency	
*DMD* (300377)	Xp21.2-p21.1	Duchenne muscular dystrophy (DMD)	XLR	59	0.052%	34	0.030%	93
		Becker muscular dystrophy (BMD)						
*HBB* (141900)	11p15.4	β-Thalassemia; Sickle cell disease	AD/AR	39	0.035%	0	0.000%	39
*PCDH15* (605514)	10q21.1	Deafness, autosomal recessive 23	AR	39	0.035%	78	0.069%	117
		Usher syndrome, type 1 F						
*F8* (300841)	Xq28	Hemophilia A	XLR	18	0.016%	47	0.042%	65
*MCPH1* (607117)	8p23.1	Primary microcephaly 1, recessive	AR	17	0.015%	20	0.018%	37
*GBE1* (607839)	3p12.2	Glycogen storage disease, type IV	AR	17	0.015%	3	0.003%	20
		GBE1-related disorders						
*USH2A* (608400)	1q41	Usher syndrome, type 2 A	AR	13	0.012%	12	0.011%	25

CNVR, copy number variant region; OMIM, Online Mendelian Inheritance in Man; AR, autosomal recessive inheritance; AD, autosomal dominant inheritance; XLR, X-linked recessive inheritance.

We identified 246 novel CNVRs, 54.47% (134/246) involving protein-coding genes (Supplementary Table S5). The median size of the novel CNVRs was 330 Kb, containing a median of 2 [0,137] protein-coding genes, indicating limited pathogenicity of most of CNVRs. On average, each duplication contained 6.55 protein-coding genes, which was more than the deletion (1.2 protein-coding genes, *P* < 0.0001).

### CNVRs in 22q11.2 region and DMD gene

To further demonstrate the clinical value of NIPS data in detecting maternal CNVs associated with disease, we analyzed the 22q11.2 region and the *DMD* gene. In our study, 7,411 CNVs containing the 22q11.2 region were detected and grouped into 130 duplication and 24 deletion CNVRs, which were shown in CNVR distribution map Figure 3(d). There are eight low-copy recurrent regions (LCRs) in 22q11.2. We found 5 cases with LCRA-B, 20 with LCRB-D deletions, and 2 with LCRE-F deletions in all 113,017 participants, with a carrier rate of 0.024% (27/113,017). No LCRA-D deletions were observed. In the 22q11.2 deletion syndrome-related dosage-sensitive key genes, we detected five deletions involving the *TBX1* gene and 21 deletions involving the *CRKL* gene, but no CNVs causing *SMARCB1* deletion were detected. Compared to deletions, duplications were more frequent. We detected 40 cases with LCRA-B, 51 with LCRB-D, 4 with LCRC-D, 2 with LCRD-E, 15 with LCRE-H, 17 with LCRE-F, and 23 with LCRG-H duplications, with a carrier rate of 0.13% (152/113,017).

As one of the largest genes, 112 deletions and 55 duplications involved the *DMD* gene, resulting in 32 deletions and 26 duplication CNVRs, which were shown in CNVR distribution map Figure 3(e). The most frequent CNVRs are from exon45 to exon55 (Supplementary Figure S7). A total of 93 participants carried CNVRs (34 duplications, 59 deletions) involving *DMD* exons, with a carrier rate of 0.082% (93/113,017). Information on CNVRs in the 22q11.2 region and *DMD* were detailed in Supplementary Tables S6 and S7.

## Discussion

In this study, we developed and validated a novel approach to investigate maternal CNV calls using NIPS data in large populations. To our knowledge, this study represents the largest cohort to date investigating maternal CNVs based on NIPS data. Several studies have performed genome-wide maternal CNV detection in small cohorts (less than 10,000 individuals) using NIPS data, with algorithm comparable to those in this work [13,14]. Maternal CNVs could be detected in these studies, but the detection of rare CNVs is limited. Benefiting from a sample size over 100,000 individuals, our study enables the identification of a broader spectrum of low-frequency variants across the whole genome. This population size is critical in improving the discovery of rare disease-associated variants [10,31]. In this regard, several ongoing projects are profiling these variants at the scale of 100,000 individuals, including the ‘all of us’ program, UK Biobank, FinnGen, the GenomeAsia 100k Project, and the Han100K [32–36]. Benefiting from the large-scale population, we also present a rare CNVR map by providing maternal care for our community. In the current study, we conducted NIPS on 113,017 pregnant women, representing approximately 5% of the local female population.

NIPS data are a reliable resource for analyzing maternal CNVs [4,14]. Our study supports this concept as we validated these CNVs using the NIPS sequencing data from over 5,000 pregnant women in two independent pregnancies. The validation showed that NIPS could call CNVs larger than 100 Kb with high consistency or confidence (95.88% for deletions and 93.75% for duplications). However, consistency is compromised for CNVs between 50 Kb and 100 Kb. This result is due to the adoption of internal calibration within the same sequencing batch to reduce data fluctuations and minimize false positive CNV calls, but at the same time, it will reduce detection sensitivity.

This study generated a large-scale regional population-based CNVR map based on 0.15× genome-wide sequencing data produced by NIPS. Compared with genomic sequencing at 3× coverage, using 0.15× sequencing coverage in this study can increase the sample size by 20-fold at the same total sequencing cost [37]. This sample size increase can be further promoted in our subsequent clinical practice of maternal care. Notably, due to the large population size, our study confirmed CNVRs previously reported in small cohorts and identified novel rare CNVRs [11].

As the smallest chromosome in women, chromosome 22 produces the most duplication CNVs, suggesting its impact on women’s health at the population level. We noticed two representative regions, 22q11.2 and the *DMD* gene, which contains several CNVs. The 22q11.2 region was associated with 22q11.2 deletion syndrome or duplication syndrome by loss or gain in several classical recurrent regions between LCR-A and LCR-H [38]. In our observation, a few pregnant women have pathogenic deletions and duplications. We did not detect the LCRA-D deletion which was reported in 70%–90% of patients with classic phenotypes [39,40]. On the contrary, only small deletions have been observed in pregnant women, which were reported weaker pathogenic effect and less impact on reproduction [41]. The carrier rates of classic pathogenic CNVs in the 22q11.2 region in this study are similar to those of a previous study based on UK biobank data by Zamariolli et al. [42] Regarding the *DMD* gene, its carrier rate of defects in pregnant women is 0.04%, mainly located in a hotspot region from exon45 to exon55, similar to the report in the Belgian population [11]. Furthermore, we detected a known hotspot deletion region in intron44 of the *DMD* gene, which was predicted to be harmless for gene function [43].

We provide insights into the impact of maternal CNVs obtained from NIPS on maternal-fetal health. By analyzing the genes involved in CNVRs, we observed that a small number of CNVRs overlap with the exons of pathogenic genes associated with dominant or XLR-inherited diseases. According to this study, approximately 0.28% of pregnant women may be carriers of CNVRs that are ‘clinically actionable’ and require further genetic counseling or laboratory confirmation. These pregnant women may be affected by complete or partial phenotypes or carriers without any phenotypes. However, their offspring may be at risk of developing disease or having more severe phenotypes. Our study shows that it is feasible to detect CNVRs overlapping genes recommended for carrier screening based on NIPS sequencing data, but how to provide further clinical interventions requires further studies. The ACMG guideline for NIPS suggests that although it is not the purpose of NIPS to identify clinically relevant maternal genomic information, patients and providers should be aware of the potential for incidental discovery of such information and additional follow-up testing unrelated to the pregnancy [44]. However, due to ethical and privacy considerations and lack of other methodological validation, we did not inform carriers of these unexpected findings in this study.

Nevertheless, this knowledge has potential clinical implications that must be considered appropriately. In addition, a distinctive polymorphism can be obtained by NIPS, which can be used as an individual identification feature in CNVs obtained from these sequencing data. The presence of CNVRs that overlap genes recommended for carrier screening also indicates that a small group of pregnant women may be carriers of a particular genetic disease. Therefore, although the ultra-low-depth sequencing data from NIPS is a sparse short-read genomic dataset, it should be handled with particular concern as it contains personal genomic information.

Some limitations of this study need to be improved in subsequent studies. First, this study is a hypothesis-generating methodological feasibility study. Without available follow-up data, definitive validation of the true clinical relevance of these CNVs cannot be performed. Further follow-up studies are required to verify clinical benefits of this workflow. Second, the breakpoints of CNVs are only approximate estimates, limited by the window size in segmentation, with an error level of 10 Kb in the genome. In addition, CNVs in chromosome X may be influenced by the sex of the fetus and contain more false calls.

## Conclusions

We generated a regional population-based female CNVR map using large-scale NIPS data and validated the reliability of the CNVs. Our results indicate that the 0.15× sequencing data from NIPS is a reliable, cost-effective, and easily accessible data source for maternal CNV detection. The maternal CNV information, as an extension of the secondary finding of NIPS, enriches the CNV database and is a novel way to conduct large-scale population research. At the same time, it provides a revealing insight into the carrier screening value of NIPS data in women’s and infants’ health care and brings new ethical and genetic counseling challenges to the application of NIPS.

## Supplementary Material

supplymentary table_20260603.xlsx

supplymentary data_20260603.docx

## Data Availability

The authors confirm that the data supporting the findings of this study are available within the article and its supplementary materials. Raw fastq data were generated and stored at Maternity & Child Healthcare Hospital of Longgang District, Shenzhen City. Access to the raw data should be granted only upon authorization from the corresponding author.
