# Supplementary material for: Identification of rare maternal copy number variants by genome-wide analysis of noninvasive prenatal screening data in 113,017 pregnant women
Source: Ann Med. 2026 Sep 9;58(1):2721711. doi: 10.1080/07853890.2026.2721711 (PMC13560419; doi:10.1080/07853890.2026.2721711)
Supplement: Supplementary file 1 [file IANN_A_2721711_SM1822.docx]

**Supplementary information for**

**Identification of rare maternal copy number variants by genome-wide analysis of non-invasive prenatal screening data in 113,017 pregnant women**

**Supplementary methods**

NIPS librarying workflow

Maternal cell-free plasma was separated from 5 ml of maternal whole blood collected in an EDTA-K2 tube by centrifugation at 1600 × g (10 min, 4°C). The plasma was centrifuged at 13000 × g (10 min, 4°C) and stored at -80°C before library preparation. DNA extraction and molecular barcode ligation were performed for each sample according to the manufacturer’s instructions for the NIFTY chromosomal abnormality test kit (BGI, China). Library qualification was determined using Qubit 3.0 (Thermo, USA). Finally, 48 single-sample libraries were pooled into one mixed library for single-end sequencing.

CNV calling using the circular binary segmentation (CBS) algorithm

Each sample's sequencing depth of 10kb segmental bins was collected for copy number variants (CNV) calling using a DNAcopy package based on the CBS algorithm(1). For the first step results of CBS, adjacent CNV calls were merged into combined ones by setting the parameter ‘undo.splits’ as ‘sdundo’ and ‘undo.SD’ as ‘3’, which undoes split CNVs within 3 standard deviations (SDs) of sequencing depth. Next, a p-value was obtained by applying Siegmund's approximation for the maximum statistic from binary segmentation of consecutive segments within a chromosome. Combined CNV calls with p-value < 0.05 were filtered as significant CNVs for subsequent analysis(2).

Data resources for CNV region (CNVR) annotation

### To access structural lists of known CNVRs in public databases, we used three dbVar collections (nstd102(3), nstd174(4), and nstd183(5)) as annotations in this study. The study of nstd102 is a case set containing clinical structural variants (SV) and submitted to the ClinVar database by external laboratories, with a submitter version of ‘2023/08’ and last updated on 2023-07-30 (<https://www.ncbi.nlm.nih.gov/dbvar/studies/nstd102/>). The study of nstd174 contains structural variants curated by the Database of Genomic Variants (DGV) as ‘Gold Standard Variants’, with a submitter version of ‘May 2016’ (<https://www.ncbi.nlm.nih.gov/dbvar/studies/nstd174/>). The study from nstd183 represents common population CNVs displayed in the DECIPHER database, with a submitter version of ‘Feb 2020’(<https://www.ncbi.nlm.nih.gov/dbvar/studies/nstd183/>). The RefSeq gene set from NCBI (National Center for Biotechnology Information) was downloaded at <https://hgdownload.soe.ucsc.edu/goldenPath/hg19/database/ncbiRefSeq.txt.gz> and last updated on 2022-03-16(6). OMIM gene set was requested from <https://omim.org/downloads>, and updated to 2020-07(7) The lists of curated dosage-sensitive genes and regions were downloaded from ClinGen( <https://ftp.clinicalgenome.org/ClinGen_gene_curation_list_GRCh37.tsv> and <https://ftp.clinicalgenome.org/ClinGen_region_curation_list_GRCh37.tsv>) , updated on 2023-10-15. Information on recurrent regions was referred to <https://ftp.clinicalgenome.org/ClinGen%20recurrent%20CNV%20.bed%20file%20V1.1-hg19.bed>, with a last update on 2021-02-02(8,9).

**Reference:**

1.	Olshen AB, Venkatraman ES, Lucito R, Wigler M. Circular binary segmentation for the analysis of array-based DNA copy number data. Biostat Oxf Engl. 2004;5:557–72.

2.	Venkatraman E. Seshan AO. DNAcopy: DNA copy number data analysis. Bioconduct Vignette. 2022.

3.	Landrum MJ, Lee JM, Benson M, Brown GR, Chao C, Chitipiralla S, et al. ClinVar: improving access to variant interpretations and supporting evidence. Nucleic Acids Res. 2018;46:D1062–7.

4.	MacDonald JR, Ziman R, Yuen RKC, Feuk L, Scherer SW. The Database of Genomic Variants: a curated collection of structural variation in the human genome. Nucleic Acids Res. 2014;42:D986-992.

5.	Firth HV, Richards SM, Bevan AP, Clayton S, Corpas M, Rajan D, et al. DECIPHER: Database of Chromosomal Imbalance and Phenotype in Humans Using Ensembl Resources. Am J Hum Genet. 2009;84:524–33.

6.	O’Leary NA, Wright MW, Brister JR, Ciufo S, Haddad D, McVeigh R, et al. Reference sequence (RefSeq) database at NCBI: current status, taxonomic expansion, and functional annotation. Nucleic Acids Res. 2016;44:D733-745.

7.	Amberger JS, Bocchini CA, Schiettecatte F, Scott AF, Hamosh A. OMIM.org: Online Mendelian Inheritance in Man (OMIM®), an online catalog of human genes and genetic disorders. Nucleic Acids Res. 2015;43:D789-798.

8.	Rehm HL, Berg JS, Brooks LD, Bustamante CD, Evans JP, Landrum MJ, et al. ClinGen--the Clinical Genome Resource. N Engl J Med. 2015;372:2235–42.

9.	Riggs ER, Nelson T, Merz A, Ackley T, Bunke B, Collins CD, et al. Copy number variant discrepancy resolution using the ClinGen dosage sensitivity map results in updated clinical interpretations in ClinVar. Hum Mutat. 2018;39:1650–9.

**Supplementary Figures**

**Figure S1.**

Distribution of individual information of 113,017 participants and distribution of sequencing depth in consistency validation. (a). Distribution of maternal age of participants; (b). Distribution of participants' gestation age at sampling for NIPS; (c). Distribution of fetal fraction of samples; (d). The relative distribution density of geographic information of participants' birthplace; (e). The distribution density of sequencing depth of consistent and inconsistent CNVs in two pregnancies of one woman.

**Figure S2.**

CNVR maps (chromosome 1-5) of pregnant women in Southern China. (i-v). Distribution of CNVR in chromosome 1-5, annotated by RefSeq genes, OMIM genes, dosage-sensitive genes, and recurrent CNV regions. Each CNVR presented as a rectangle, with left and right borders indicating the upstream and downstream breakpoints. The darkness of color represents the frequency of CNVRs; the darker the color, the higher the frequency. DS: dosage-sensitive; BP: breakpoint; AR: autosomal recessive inheritance; AD: autosomal dominant inheritance; XLR: X-linked recessive inheritance; XLD: X-linked dominant inheritance.

**Figure S3.**

CNVR maps (chromosome 6 -10) of pregnant women in Southern China. (i-v). Distribution of CNVR in chromosome 6-10, annotated by RefSeq genes, OMIM genes, dosage-sensitive genes, and recurrent CNV regions. Each CNVR presented as a rectangle, with left and right borders indicating the upstream and downstream breakpoints. The darkness of the color represents the frequency of CNVRs; the darker the color, the higher the frequency. DS: dosage-sensitive; BP: breakpoint; AR: autosomal recessive inheritance; AD: autosomal dominant inheritance; XLR: X-linked recessive inheritance; XLD: X-linked dominant inheritance.

**Figure S4.**

CNVR maps (chromosome 11-15) of pregnant women in Southern China. (i-v). Distribution of CNVR in chromosome 11-15, annotated by RefSeq genes, OMIM genes, dosage-sensitive genes, and recurrent CNV regions. Each CNVR presented as a rectangle, with left and right borders indicating the upstream and downstream breakpoints. The darkness of the color represents the frequency of CNVRs; the darker the color, the higher the frequency. DS: dosage-sensitive; BP: breakpoint; AR: autosomal recessive inheritance; AD: autosomal dominant inheritance; XLR: X-linked recessive inheritance; XLD: X-linked dominant inheritance.

**Figure S5.**

CNVR maps (chromosome 16-20) of pregnant women in Southern China. (i-v). Distribution of CNVR in chromosome 16-20, annotated by RefSeq genes, OMIM genes, dosage-sensitive genes, and recurrent CNV regions. Each CNVR presented as a rectangle, with left and right borders indicating the upstream and downstream breakpoints. The darkness of the color represents the frequency of CNVRs; the darker the color, the higher the frequency. DS: dosage-sensitive; BP: breakpoint; AR: autosomal recessive inheritance; AD: autosomal dominant inheritance; XLR: X-linked recessive inheritance; XLD: X-linked dominant inheritance.

**Figure S6.**

CNVR maps (chromosome 21,22 and X) of pregnant women in Southern China. (i-iii). Distribution of CNVR in chromosome 21,22, and X, annotated by RefSeq genes, OMIM genes, dosage-sensitive genes and recurrent CNV regions. Each CNVR presented as a rectangle, with left and right borders indicating the upstream and downstream breakpoints. The darkness of the color represents the frequency of CNVRs; the darker the color, the higher the frequency. DS: dosage-sensitive; BP: breakpoint; AR: autosomal recessive inheritance; AD: autosomal dominant inheritance; XLR: X-linked recessive inheritance; XLD: X-linked dominant inheritance.

**Figure S7.**

Distribution of affected exons of the *DMD* gene in 113,017 pregnant women. Deletions in a hotspot ranging from exons 45 to 55, known as the ‘Exon 45-55 skipping’ region, are shown as orange bars. CNVR: copy number variants region.

**Legends for Supplementary tables**

**Table S1. CNVRs with frequency over 1%**

**Table S2. CNVRs that overlapped known pathogenic or likely pathogenic regions and with frequency over 0.01%**

**Table S3. CNVRs that located in known recurrent regions and with frequency over 0.01%**

**Table S4. Dosage-sensitive genes that overlapped CNVRs with frequency over 0.01%**

**Table S5. Novel CNVRs that overlapped RefSeq genes**

**Table S6. CNVRs in 22q11.2 region**

**Table S7. CNVRS in *DMD* gene**
